# Detection and Molecular Characterization of *Blastocystis* Species in Polish Soldiers Stationed in the Republic of Kosovo

**DOI:** 10.3390/ijms241814100

**Published:** 2023-09-14

**Authors:** Barbara Pietrzak-Makyła, Krzysztof Korzeniewski, Paweł Gładysz, Anna Lass

**Affiliations:** 1 Department of Tropical Parasitology, Institute of Maritime and Tropical Medicine in Gdynia, Medical University of Gdańsk, Powstania Styczniowego 9b, 81-519 Gdynia, Poland; baspie007@gmail.com (B.P.-M.); pawel.gladysz@gumed.edu.pl (P.G.); 2 Department of Epidemiology and Tropical Medicine, Military Institute of Medicine—National Research Institute, Szaserów 128, 04-141 Warsaw, Poland; kkorzeniewski@wim.mil.pl

**Keywords:** *Blastocystis*, PCR, subtypes, military environment, Kosovo

## Abstract

*Blastocystis* species (sp.) is one of the less well-understood water- and foodborne protozoa of medical and veterinary importance linked to different gastrointestinal disorders. Soldiers participating in military missions are particularly vulnerable to infection with this protozoa. The present study used molecular methods to detect, identify, and subtype (ST) *Blastocystis* sp. in Polish soldiers stationed in the Republic of Kosovo. Fecal samples were collected from 192 soldiers on arrival and after four months of stay. After DNA extraction, the barcoding region of the small subunit ribosomal RNA (SSU-rRNA) gene was amplified and sequenced. The DNA of *Blastocystis* sp. was detected in six (3.13%) and thirty (15.16%) samples in the first and second batch, respectively. Sequencing analysis revealed infections with ST 2, 3, 4, and 7. There was no statistical association between *Blastocystis* sp. infection and the parasite’s ST or the age or rank of soldiers. The results indicate that the visit to a new environment and prolonged stay in the area of military operation in Kosovo resulted in a significant increase in both *Blastocystis* sp. infections and ST diversity among surveyed soldiers. This shows the need to undertake appropriate countermeasures to reduce *Blastocystis* infections in the military environment abroad.

## 1. Introduction

*Blastocystis* species (sp.) is a eukaryotic protist distributed worldwide, occurring in the intestines of humans and various animals, including primates, mammals, birds, reptiles, amphibians, and insects [1,2]. It has been estimated that as many as one billion people can be infected with this microorganism. Its prevalence differs between geographical regions, countries, and communities and may reach from 2.5% to 56% in developed countries and even up to 100% in developing countries [3,4,5,6]. Multiple risk factors may play a role in the distribution of *Blastocystis* sp. infection, most importantly, the level of sanitation infrastructure and hygiene habits but also the diversity of studied populations, such as age, health and nutritional status, place of residence (village/town), and habits and lifestyle (contact with animals, not washing hands after using toilets, drinking unfiltered water) [7,8]. It is presumed that infection is acquired via the fecal–oral route, although the life cycle of *Blastocystis* sp. is not yet fully understood.

The pathogenicity of *Blastocystis* sp. and its importance to public health are controversial since the organism is found in both asymptomatic and symptomatic individuals [9]. In the latter group, a wide range of gastrointestinal disorders have been observed, from acute watery diarrhea to mild chronic abdominal discomfort (abdominal pain, nausea, bloating, constipation, flatulence). Some researchers have also linked *Blastocystis* sp. infection to irritable bowel syndrome (IBS) and irritation bowel disease (IBD), as well as to some extraintestinal symptoms, such as headache, chronic fatigue, itching, and skin rash [10,11,12,13]. The extent and severity of symptoms may be connected with the pathogenicity of the strain as well as the immunological status of the infected person [6,14,15].

*Blastocystis* sp. is delicate, easily destructible, and highly polymorphic, which manifests itself in the varied appearance and size range of its existing forms [14,16]. This makes routine microscopic examination of stool difficult and often unreliable. Fortunately, the recent development of different molecular methods has not only significantly improved the sensitivity and specificity of *Blastocystis* sp. detection in stool samples but also has made it possible to determine its subtypes (STs) [3,16,17,18].

*Blastocystis* sp. shows extensive genetic diversity within the small subunit of the ribosomal RNA (SSU-rRNA) gene [19], which allowed researchers to identify several microscopically indistinguishable STs [20]. Ten (STs 1–9 and ST 12) are found in humans, but up to 95% of human infections are caused by STs 1–4 [1,2,14,21,22]. Nonetheless, a possible correlation between a particular ST and its pathogenic potential is still strongly debated [1,7].

Armed conflicts have been relatively frequent in recent years, especially in the Middle East. Soldiers deployed on military and peacekeeping operations in developing countries are at higher risk of contracting infectious diseases, including intestinal parasitic infections, due to difficult sanitary and hygienic conditions [23,24,25,26]. Only a few studies show the prevalence of *Blastocystis* sp. infection in troops. None of them, however, are based on molecular assay.

This study aimed to estimate the level of infections with *Blastocystis* sp. and determine the genetic diversity of the detected parasites in Polish soldiers participating in a peacekeeping mission in the Republic of Kosovo, using molecular methods.

## 2. Results

The barcoding fragment for *Blastocystis* sp. was successfully amplified in six (3.13%) samples investigated in the study group upon arrival and thirty (15.6%) samples investigated in the same study group after four months of stay (Table 1, Figure S1). Sequencing and ST determination was successful in 26 out of 36 positive samples obtained in total. The sequences were deposited in GenBank (accession nos.: OR062372–OR062375 and OR062551–OR062572). In the case of ten samples, including two collected in the first and eight collected in the second round of studies, *Blastocystis* sp. STs could not be identified due to a PCR product concentration that was insufficient for successful sequencing (Table 1).

Four different STs of *Blastocystis* sp. were detected, namely ST 2, 3, 4, and 7 (Table 1). Among the four STs, the most frequently detected was *Blastocystis* sp. ST 3 both in the first and second round of research (Table 1). Its occurrence was determined in four out of six (66.66%) positive isolates in the first batch of samples and in eleven out of thirty (36.66%) positive samples tested in the second batch. In the second set of samples, STs 2 (16.66%), 4 (10%), and 7 (10%) were also detected apart from ST 3.

**Figure 1:**
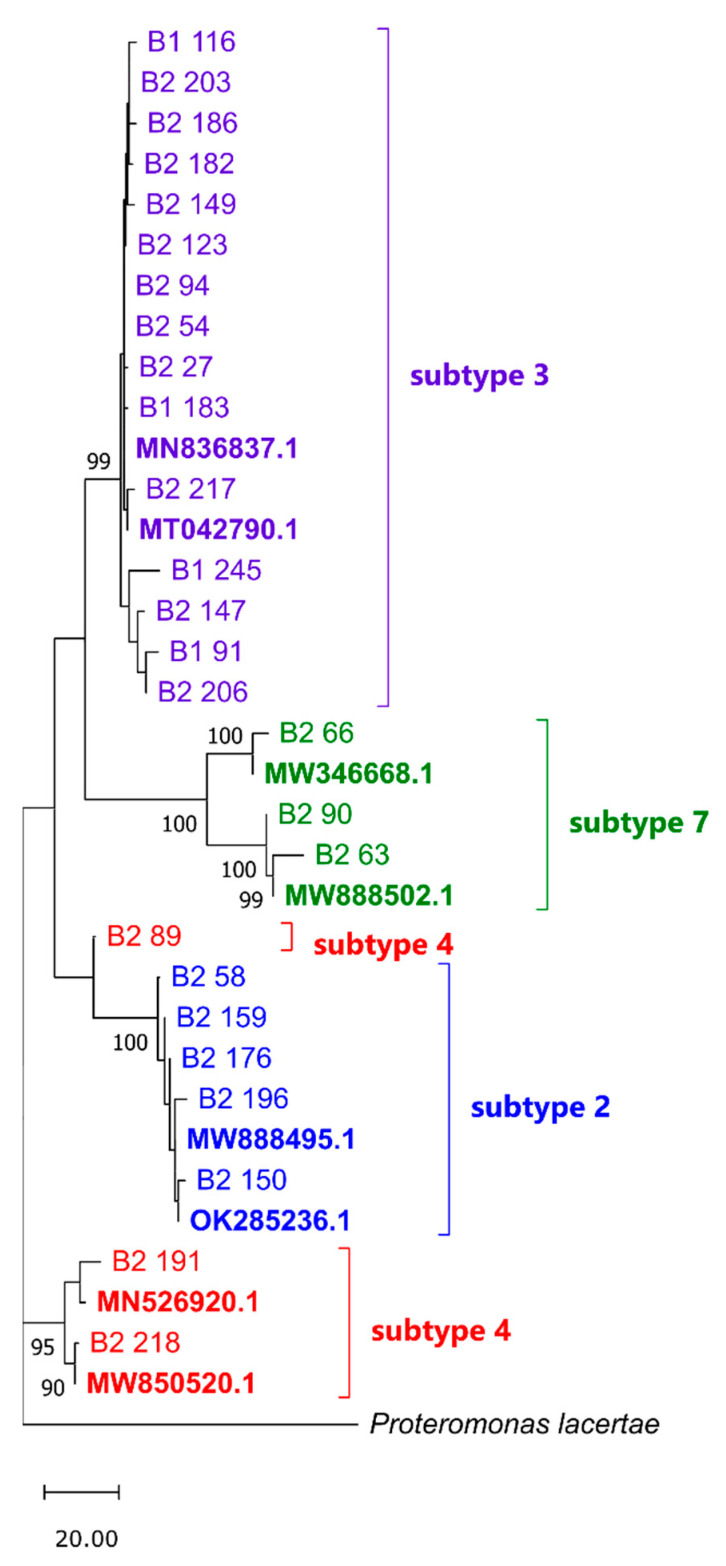
Tree no. 1 out of the four most parsimonious trees (length = 292) generated for positive samples with established *Blastocystis* sp. subtype and GenBank sequences of known subtype (accessions in bold). The consistency index is 0.845890 (0.763158), the retention index is 0.924115 (0.924115), and the composite index is 0.781700 (0.705245) for all sites and parsimony-informative sites (in parentheses). Clustering was assessed in the bootstrap test (1000 replicates; values > 80% are shown below the branches). The tree is drawn to scale, with branch lengths calculated using the average pathway method and in the units of the number of changes over the whole sequence. B1—first batch of samples, B2—second batch of samples.

The groupings were supported by the phylogenetic analysis. The Maximum Parsimony tree revealed monophyletic clusters of sequences identified as STs 2, 3, and 7 with a strong bootstrap support (BS) ≥99% (Figure 1). The phylogeny of the three ST-4 samples was weakly resolved due to the shortness of sequence 89 (248 bp), resulting in a polyphyletic arrangement where samples 191 and 218 clustered together (BS = 95%), and sample 89 grouped with ST-2 samples (BS = 63%).

Regarding the soldiers’ age, the distribution of *Blastocystis* sp. infection was as follows: <25 years—two (15.38%) positive isolates detected only among soldiers tested in the second round of research; 26–35 years—four (4.7%) and fifteen (17.65%) positive isolates detected among soldiers tested in the first and the second round of research, respectively; 36–45 years—two (2.47%) and eleven (13.58%) positive isolates detected among soldiers tested during the first and the second round of research, respectively; >46 years—two (15.38%) positive isolates detected only among soldiers tested in the second round of research (Table 1). Based on the data for the second part of the stationing, the youngest *Blastocystis*-positive soldier was 24 years old, and the oldest was 50 years old (median: 32 years of age). There was no statistically significant relationship between the soldiers’ age and *Blastocystis* sp. ST in the Kruskal–Wallis test (H(3) = 0.6006608, *p* = 0.8963).

The distribution of *Blastocystis* sp. infection by military rank was as follows: privates—three (4%) positive samples detected among soldiers tested during the first round of research and eleven (14.66%) positive samples detected among soldiers tested in the second round of research, respectively; non-commissioned officers—three (3.41%) and twelve (13.63%) positive samples detected among soldiers tested during the first and the second round of research, respectively; officers—six (20.69%) positive isolates detected only among soldiers tested in the second round of research (Table 1). Pearson’s chi-square independence test showed no statistically significant relationship between the rank and *Blastocystis* sp. ST (Χ^2^ = 3.386243, df = 6, *p* = 0.75904). ST 3 was the most common variant among all ranks, with a prevalence of 55.56% in privates, 50% in officers, and 42.86% in non-commissioned officers. ST 7 was not detected in privates.

Out of six soldiers positive for *Blastocystis* sp. upon arrival, only one was still positive after four months of stay in Kosovo. Interestingly, two different subtypes were detected in this soldier in the first (ST 3) and second (ST 2) round of testing (Table S1).

## 3. Discussion

Soldiers deployed on military operations in developing countries are at a higher risk of contracting gastrointestinal infections. Despite that, only a few articles are devoted to analyzing parasitic intestinal infections among soldiers stationed abroad on military bases. Most focus on detecting common pathogenic protozoan parasites and helminths without considering *Blastocystis* sp. [23]. As for Polish contingents, the examination of Polish soldiers deployed to Chad and Central Africa in 2008–2009 demonstrated a high rate of intestinal parasitic infections, predominantly with *Giardia intestinalis* (22.3%) [25]. Similar results were obtained from examinations of the Polish Military Contingent (PMC) members deployed to Afghanistan in 2011 [25]. The only study where soldiers were tested for *Blastocystis* sp. was performed in 2008–2010 and concerned missions in Afghanistan and Iraq. Stool samples were collected from 913 Polish soldiers twice, before departure and on return, and investigated by direct smear examination. Some of the examined samples contained vacuolar forms of *Blastocystis* sp. (15.3%) [24]. Microscopic examination, however, was not subsequently verified by molecular methods, so *Blastocystis* sp. STs were not determined.

In this study, we focused on the prevalence of *Blastocystis* sp. infection among soldiers of the PMC deployed on the Kosovo Force (KFOR) carrying out a stabilization mission in the Republic of Kosovo. A group of Polish soldiers stationed in the international base in Novo Selo was tested for the presence of the parasite twice, on arrival and after four months of stay, using molecular methods. Results of the studies showed a significant, five-fold increase in infections. Moreover, this increase was observed in all the age groups studied and in each military rank. We found no correlation between age or rank and the ST of *Blastocystis* sp. that caused the infection. This aligns with the results obtained by Duda [24] in Afghanistan and Iraq.

Developing countries have a higher prevalence of *Blastocystis* sp. than industrialized countries. Since the fecal–oral route is considered to be the primary mode of transmission of this parasite [3], the most important factors influencing the number of infections in humans are poor hygiene practices, close animal contact, and consumption of contaminated food or drinking of contaminated water [27,28,29,30,31,32,33,34]. When considering the reasons for the increase in the number of infected soldiers during their stay at the base, it is necessary to consider both internal and external risk factors.

Soldiers from Novo Selo interacted with local communities during the peacekeeping mission. They were patrolling the area accompanied by the Kosovo Police or they participated in joint synchronized border patrols with soldiers of the Serbian army. They also had opportunities to meet local inhabitants while participating in humanitarian aid operations, including visits to local administration, schools, and health centers. According to our preliminary study of the population living in Kosovo, at least 26% of tested inhabitants were *Blastocystis*-positive. This indicates a high chance of the soldiers becoming infected through contact with the Kosovar population. Additionally, apart from the fresh meals served at the base made mainly from produce imported by the KFOR, soldiers could eat at local restaurants outside the base and consume local produce which could also be an important source of infection. Therefore, there is a high probability that *Blastocystis* sp. transmission could result from contact with local people and/or food. On the other hand, some soldiers from the maneuvering platoons participated in outdoor training that involved spending several weeks in the field, which additionally may have exposed them to parasitic infection due to poor hygienic conditions. Nonetheless, the above strongly suggests the possibility of contracting *Blastocystis* sp. by Polish soldiers while performing activities outside the military base.

To date, there are no data about the prevalence of *Blastocystis* sp. in the population of Kosovo, and very few studies refer to the presence of this microorganism in the inhabitants of neighboring countries. For example, in Serbia, 50 fecal samples from patients with gastrointestinal disorders were found positive for *Blastocystis* sp. during a routine microscopic investigation performed by the Public Health Institute Niš between 2012 and 2016 [35]. However, the authors of the publication did not provide information about the total number of individuals tested, so it is impossible to estimate the overall percentage of those infected with *Blastocystis* sp. Nonetheless, subsequent molecular studies of human fecal samples revealed the presence of *Blastocystis* sp. ST 3 and ST 6 alongside ST 5 in pigs. In another study performed in 2005 in Albania, 277 fecal samples collected from random healthy people visiting public health centers in the city of Mamuras were tested for intestinal protozoa using microscopic methods. Infection with *Blastocystis* sp. was the most prevalent and reached 45%; STs were not determined [36]. *Blastocystis* sp. was also isolated from 42 (16.09%) out of 261 patients hospitalized at the University Hospital in Pleven, Bulgaria. Isolates were identified as ST 1 and ST 3 [37]. A study of 73 patients with irritable bowel syndrome and colitis hospitalized in Iași, Romania, revealed STs 1, 2, and 4; interestingly, ST 2 predominated [38]. Although data on *Blastocystis* sp. in the Balkan countries surrounding Kosovo are sparse, such data show a high rate of infection among the local population, and it is highly probable that the epidemiological situation in Kosovo is similar.

At the beginning of their stay in the Novo Selo base, the Polish soldiers investigated in this study were infected only with *Blastocystis* sp. ST 3, which is the main ST in Poland [39]. After four months, ST 3 was still dominant, but three additional STs, namely 2, 4, and 7, were also noted. The barcoding fragment of the 5′ end of the SSU-rRNA gene recommended for unambiguous subtyping measures approx. 600 bp, and the vast majority of analyzed sequences met this requirement. The two shorter sequences obtained for samples 89 and 245 were sufficient to identify the isolate [40]. However, we treat these two results with caution. The ST of 10 *Blastocystis*-positive samples could not be identified due to the concentration of DNA insufficient for sequencing. 

Scarce data on the prevalence of *Blastocystis* sp. and its variants in Kosovo and neighboring countries make the interpretation of our results difficult. ST 3 is the most common variant in humans, accounting for up to 44% of infections [41], and it is probably of human origin. Large-scale human-to-human transmission explains its predominance in humans [13,42,43]. Therefore, it is not surprising that ST 3 was significantly overrepresented in the investigated soldiers. The soldiers most likely contracted *Blastocystis* sp. either from each other at the base or from Kosovan residents outside. In our study, ST 2 was the second most frequently detected genetic profile in the soldiers. According to available literature, in most cases, it was less often detected than ST 1 and ST 3 or not noted at all [41,44]. However, data from South American countries and Romania show the opposite trend [21,38]. Our pilot studies among local Kosovan communities confirm that ST 2 is the second most frequent *Blastocystis* variant after ST 3 which reinforces contact with the local community as an important risk factor. ST 4 and ST 7 showed the same frequency in the investigated soldiers. ST 7 is slightly more often detected in Asia than Europe [22,41] and is considered relatively rare in humans. This subtype prefers avian hosts [2,41,45,46], but it has also been detected in Bovidae, carnivores, and non-human primates [46]. Interestingly, the presence of ST 7 was not confirmed in studies performed in other Balkan countries surrounding Kosovo, so it is hard to speculate about the source of the infection. ST 4 is rarely detected outside Europe [41] but was previously found in the Romanian population [38]. Polish soldiers were stationed with the Hungarian contingent. Thus, it cannot be ruled out that certain *Blastocystis* STs had been exchanged between Polish and Hungarian soldiers during their shared stay. However, it is hard to speculate what was the actual input of Hungarian soldiers into the spread of *Blastocystis* sp. in the base due to the lack of relevant data on the prevalence of the parasite in the Hungarian military or civilians.

## 4. Materials and Methods

### 4.1. Study Population

**Figure 2:**
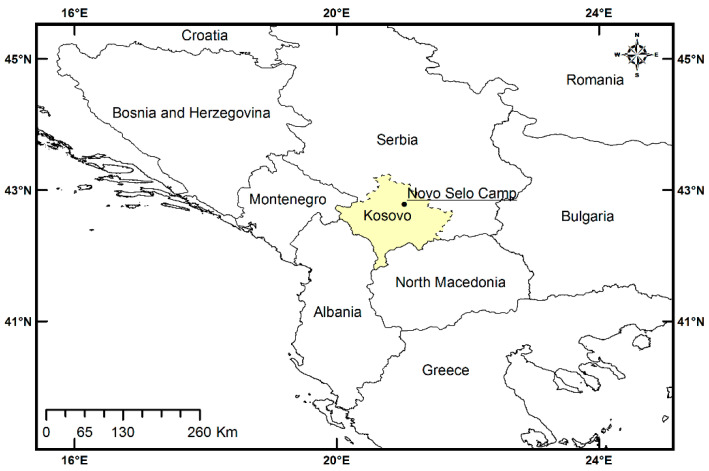
Sampling site. Map of the Republic of Kosovo with the approximate location of the international base in Novo Selo marked. Map was prepared with the use GADM data version 3.6.

In total, 192 men aged 24–55, Polish soldiers participating in a peacekeeping mission in the Republic of Kosovo, were tested for *Blastocystis* sp. in the period of November 2020–February 2021. They were part of the personnel of the Polish Military Contingent (PMC) deployed on the Kosovo Force (KFOR) operation in the northern part of Kosovo, including the neighboring Serbian municipalities of Mitrovica, Leposavić, Zubin Potok, and Zvečan (Figure 2).

Operational activities of the PMC KFOR involve performing tasks for the Multinational Battle Group—East. The soldiers investigated in this study were stationed in the international base in Novo Selo (Figure 2). All study participants were in a good general condition confirmed by medical tests and examination by a medical board before deployment to Kosovo, which was a prerequisite for military service abroad. The soldiers were not subjected to anti-parasitic prophylaxis, neither before nor during the departure. The subjects enrolled in the study were patrol, sentry, and operational troopers who frequently contacted local people and often consumed local food. The study involved soldiers who had been serving in Kosovo for a period of at least four months and had never been diagnosed with or treated for parasitic infections.

### 4.2. Sampling

Biological material was collected in the mission area by healthcare workers of the PMC. In total, 384 fecal samples were collected, two from each individual investigated. The first set of fecal samples was collected from soldiers at the beginning of their shift, and the second set of fecal samples was collected after four months of stay.

Fecal material was placed in sterile stool containers and fixed in 70% ethanol. All samples were transported to the Military Institute of Medicine in Poland and then to the Department of Tropical Parasitology, Medical University of Gdańsk.

Written informed consent was provided by all volunteers involved in the study.

### 4.3. Molecular Investigation of the Fecal Samples

#### 4.3.1. Washing of Stool Samples

To remove ethanol, each stool sample was centrifuged for 10 min at 2500× *g*, and the supernatant was carefully removed. Next, the sample was washed three times with sterile water as follows: 0.50 g of feces was placed in a sterile 2 mL tube, filled with sterile water, mixed on an automatic vortex for 20 s, and centrifuged for 3 min at 2500× *g*. The supernatant was removed with a pipette. The final pellet was stored at −20 °C for further analysis.

#### 4.3.2. Extraction of DNA from Fecal Samples

Before DNA extraction, the material was subjected to three cycles of freezing at –70 °C and thawing at 30 °C in a water bath to destroy the cyst wall and improve the efficiency of DNA extraction. Genomic DNA was then extracted from approximately 100 mg of a fecal sample using the Genomic Mini AX Stool kit (A&A Biotechnology, Gdansk, Poland) according to the original protocol. All of the PCR templates were also treated with the Anti-Inhibitor Kit (A&A Biotechnology, Gdansk, Poland), which removes polyphenolic PCR inhibitors using specific absorption particles, thereby removing factors that could interfere with the PCR. The extracted DNA was stored at −20 °C for further analysis.

#### 4.3.3. DNA Amplification

Specific detection of *Blastocystis* sp. was achieved by a direct PCR targeting the SSU-rRNA gene of the parasite [47,48]. A PCR product of approximately 600 bp (the barcode region) was amplified using BhRDr (5′-GAGCTTTTTAACTGCAACAACG-3′) and RD5 (5′-ATCTGGTTGATCCTGCCAGT-3′) primers. The 25 μL amplification reaction mixture comprised 12.5 μL of PCR Mix Plus HGC (A&A Biotechnology, Gdansk, Poland) containing recombinant Taq polymerase, PCR buffer, magnesium chloride, nucleotides, stabilizers, and gel loading buffer; 0.4 μM of each primer (Metabion, Germany); and 2 μL of template DNA.

Amplifications were performed with an initial denaturation step (4 min at 95 °C), followed by 35 cycles of denaturation (30 s at 96 °C), annealing of primers (30 s at 60 °C) and strand extension (30 s at 72 °C), and final extension (5 min at 72 °C) in a GeneAmp PCR System 9700 Thermal Cycler (Applied Biosystems, Waltham, MA, USA).

All experiments were performed with a *Blastocystis*-positive control (genomic DNA extracted from a sample successfully sequenced for *Blastocystis* sp. prior to this study) to ensure the correct functioning of the reaction, and negative controls (water template) to check the PCR components for contamination. PCR products were analyzed using the Essential V6 imaging platform (Uvitec, Cambridge, UK) following electrophoresis in agarose gel (1.5%) stained with Midori Green DNA Stain (Nippon Genetics Europe GmbH, Düren, Germany). All of the negative samples were re-tested for the presence of PCR inhibitors by mixing 2 μL of DNA template and 1 μL of *Blastocystis* sp. positive control.

#### 4.3.4. Determination of *Blastocystis* sp. STs and Phylogenetic Analysis

To determine *Blastocystis* sp. STs, all PCR products positive for *Blastocystis* sp. were purified from gels using the Gel-Out kit (A&A Biotechnology, Poland) and Sanger-sequenced bidirectionally (Macrogen, Amsterdam, The Netherlands) following a standard procedure. All obtained sequences were analyzed in GeneStudio Pro Software (GeneStudio 2.2 Inc., Suwanee, GA, USA) and subjected to Basic Local Alignment Search Tool (BLAST) searches in the GenBank database to confirm their identity. The PCR results were considered positive if direct sequencing of the products confirmed *Blastocystis* sp.

*Blastocystis* sp. subtypes (STs) were determined by querying the Public Databases for Molecular Typing and Microbial Genome Diversity (PubMLST, “www.pubmlst.org/organisms/blastocystis-spp (accessed on 30 May 2023)”) recommended by Stensvold [18], in accordance with the consensus terminology for *Blastocystis* sp. STs established in 2007 [40]. PubMLST service for *Blastocystis* sp. ST identification first checks the query against a locus or combination of loci to find an exact match. In the absence of one, the algorithm identifies the nearest partial match.

ST identification was supplemented with a phylogenetic inference using the Maximum Parsimony method in MEGA11 [49]. Sequences of varied lengths of 248 bp–598 bp were aligned using the MUSCLE algorithm with the outgroup sequence of *Proteromonas lacertae* (acc. no. U37108) and GenBank sequences of known subtype (acc. nos.: MN836837.1 and MT042790.1 for ST 3, MW346668.1 and MW888502.1 for ST 7, MW888495.1 and OK285236.1 for ST 2, MN526920.1 and MW850520.1 for ST 4) to a final set of 35 sequences containing 652 positions, including gaps. The MP tree was obtained using the Subtree–Pruning–Regrafting algorithm with search level 1 in which the initial trees were obtained by the random addition of sequences (10 replicates) (Figure 1).

### 4.4. Statistical Analysis

Statistical analysis was performed in STATISTICA Zestaw Plus version 5.0.96. (StatSoft Polska Sp. z o. o., https://www.statsoft.pl/ (accessed on 3 April 2023)). Only data from the second part of the stationing, for positive soldiers with identifiable *Blastocystis* sp. ST, were included (a total of 22 individuals).

## 5. Conclusions

One of the most important challenges for the medical base of military service abroad is to reduce the risk of soldiers being infected with intestinal parasites. They are one of the main problems of the participants of military missions, especially those stationed in countries where climatic conditions are changeable and hygienic and sanitary conditions are low, such as Kosovo. Results of our studies clearly show an increase in both the number of *Blastocystis* infections and the diversity of *Blastocystis* subtypes in tested soldiers after four months of military service in Kosovo. This indicates that the conditions on the mission are conducive to the spread of *Blastocystis* infection among the participants, and thus countermeasures should be considered in the future.

## Figures and Tables

**Table 1:** Prevalence of *Blastocystis* sp. infection in Polish soldiers participating in a peacekeeping mission in the Republic of Kosovo and distribution of *Blastocystis* sp. subtypes by rank and age. NCO—non-commissioned officer, NI—not identified, (-) not applicable.

	First Batch						Second Batch					
	Number of Positive Samples/Number of Investigated Samples (% of Positive Samples)	ST2	ST3	ST4	ST7	NI	Number of Positive Samples/Number of Investigated Samples (% of Positive Samples)	ST2	ST3	ST4	ST7	NI
**Total**	6/192 (3.13%)	0	4	0	0	2	30/192 (15.60%)	5	11	3	3	8
**Results by rank**												
**NCO**	3/88 (3.41%)	0	1	0	0	2	12/88 (13.63%)	1	3	1	2	5
**Officer**	0/29	―	―	―	―	―	6/29 (20.69%)	1	3	1	1	1
**Private**	3/75 (4.00%)	0	3	0	0	0	11/75 (14.66%)	3	5	1	0	2
**Results by age**												
**>46**	0/13	―	―	―	―	―	2/13 (15.38%)	1	1	0	0	0
**36–45**	2/81 (2.47%)	0	2	0	0	0	11/81 (13.58%)	1	1	1	0	7
**25–35**	4/85 (4.70%)	0	2	0	0	2	15/85 (17.65%)	1	9	2	3	1
**<25**	0/13	―	―	―	―	―	2/13 (15.38%)	2	0	0	0	0

## Data Availability

The sequences of Blastocystis sp. Obtained in this study are deposited in GenBank (accession nos.: OR062372; OR062373; OR062374; OR062375 and OR062551; OR062552; OR062553; OR062554; OR062555; OR062556; OR062557; OR062558; OR062559; OR062560; OR062561; OR062562; OR062563; OR062564; OR062565; OR062566; OR062567; OR062568; OR062569; OR062570; OR062571; OR062572). Other data presented in this study are available upon request from the corresponding author.

## Supplementary Materials

The supporting information can be downloaded at: https://www.mdpi.com/article/10.3390/ijms241814100/s1.
