# Supplementary material for: Detection and Molecular Characterization of Blastocystis Species in Polish Soldiers Stationed in the Republic of Kosovo
Source: Int J Mol Sci. 2023 Sep 14;24(18):14100. doi: 10.3390/ijms241814100 (PMC10531639; doi:10.3390/ijms241814100)
Supplement: Supplementary file 1 [file ijms-24-14100-s001.zip › ijms-2482620-supplementary.pdf]

Suppelmentary materials

Table S 1. Comparison of positive results of *Blastocystis* PCR obtained for stool samples collected from Polish soldiers participating in a peacekeeping mission in the Republic of Kosovo upon arrival (first part) and after four months of stay (second part).

Template no. (first/second batch)	Rank	Age	First batch of samples		Second batch of samples	
			PCR result	Subtype	PCR result	Subtype
151/130	NCO	36	negative	n/a	positive	NI
242/124	Officer	42	negative	n/a	positive	NI
14/89*	Officer	30	negative	n/a	positive	ST4
75/196	NCO	50	negative	n/a	positive	ST2
37/186	NCO	45	negative	n/a	positive	ST3
50/165	NCO	43	negative	n/a	positive	NI
137/121	NCO	43	negative	n/a	positive	NI
143/27	Officer	29	negative	n/a	positive	ST3
40/150	Officer	45	negative	n/a	positive	ST2
95/90	NCO	34	negative	n/a	positive	ST7
54/206	NCO	34	negative	n/a	positive	ST3
119/122	NCO	41	negative	n/a	positive	NI
115/172	Private	40	negative	n/a	positive	NI
44/94	Private	35	negative	n/a	positive	ST3
183/92	Private	32	positive	ST3	positive	NI
192/159	Private	25	negative	n/a	positive	ST2
47/66	Officer	28	negative	n/a	positive	ST7
33/203	NCO	47	negative	n/a	positive	ST3
28/149	Private	30	negative	n/a	positive	ST3
74/217	Private	26	negative	n/a	positive	ST3
35/182	Officer	29	negative	n/a	positive	ST3

128/147	Officer	28	negative	n/a	positive	ST3
88/218	NCO	33	negative	n/a	positive	ST4
177/138	NCO	44	negative	n/a	positive	NI
197/54	Private	29	negative	n/a	positive	ST3
194/58	Private	24	negative	n/a	positive	ST2
141/191	Private	38	negative	n/a	positive	ST4
135/26	NCO	34	positive	NI	negative	n/a
168/123	Private	26	negative	n/a	positive	ST3
245*/176	Private	36	positive	ST3	positive	ST2
46/63	NCO	33	negative	n/a	positive	ST7
91/25	Private	32	positive	ST3	negative	n/a
94/83	NCO	34	positive	NI	negative	n/a
116/102	NCO	40	positive	ST3	negative	n/a

NCO – non-commissioned officer, NI – not identified, n/a – not applicable, (*) – short PCR product (sample 89 – 248 bp, sample 245 – 289 bp)

A

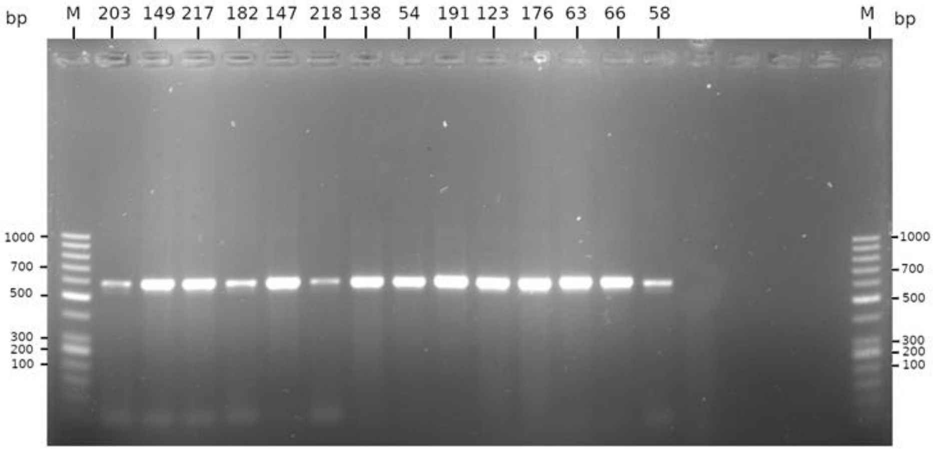

B

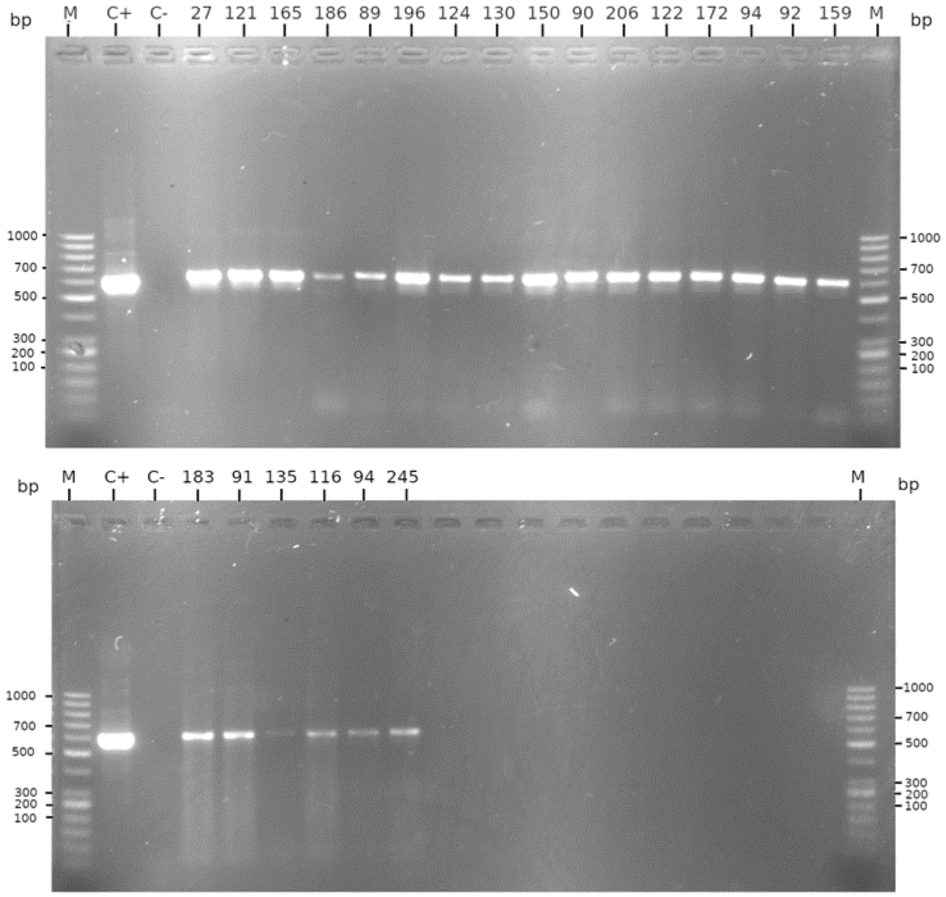

Figure S1. Positive results of PCR detection of Blastocystis sp. DNA in stool samples collected from Polish soldiers of the Polish Military Contingent stationed in Kosovo. (A) Results for the first batch of samples (soldiers tested upon arrival). M – molecular weight marker (GeneRuler 50 bp DNA Ladder, Thermo Scientific), C(+) – positive control, C(-) – negative control. Numbers 183, 135, 245, 91, and 116 represent positive samples. (B) Positive results in the second batch of samples (soldiers tested after four

months of stay). M – molecular weight marker (GeneRuler 50 bp DNA Ladder, Thermo Scientific), C(+) – positive control, C(-) – negative control.
